# Hazard ratios and their causal interpretation: challenges and guidance

**DOI:** 10.1093/ije/dyag228

**Published:** 2026-10-24

**Authors:** Jonathan W Bartlett, Dominic Magirr, Tim P Morris

**Affiliations:** Department of Medical Statistics, London School of Hygiene & Tropical Medicine, London, United Kingdom; Statistical Methodology, Novartis Pharmaceuticals AG, Basel, Switzerland; Statistical Methodology, Novartis Pharmaceuticals UK Ltd, London, United Kingdom

**Keywords:** hazard ratios, frailty, causal effect

## Abstract

The hazard ratio, typically estimated using Cox’s famous proportional hazards model, is the most common effect measure used to describe the association or effect of a covariate on a time-to-event outcome. In recent years, the hazard ratio has been argued by some to lack a causal interpretation, even in randomized trials, and even if the proportional hazards assumption holds. This is concerning, not least due to the ubiquity of hazard ratios in analyses of time-to-event data. We review these critiques and describe how we think hazard ratios should be interpreted. In light of the issues involved, alternative measures may be preferable to describe effects of exposures or treatments on time-to-event outcomes.

Key Messages

- Interpretation of hazards and hazard ratios comparing treatment or exposure groups is complicated by the possible effects of unmeasured (frailty) variables on event times.
- Hazard ratios estimated from randomized trials or from observational studies with sufficient confounder adjustment have a causal interpretation at a population level, but this interpretation should consider the possible effects of such unmeasured frailty variables.
- Other effect measures, such as contrasts of survival or event probabilities or comparisons of restricted mean survival to key landmark times of interest may be preferable to hazard ratios for communicating treatment or exposure effects for time-to-event outcomes.

Terminology Box

**Table:** 

**Term**	**Meaning**
Baseline hazard function	In a regression model for a time-to-event outcome, the hazard function for individuals for whom the model’s covariates all take value zero.
Non-collapsible	An effect measure (e.g. hazard ratio) is non-collapsible when the marginal effect differs from the conditional effects, even when the conditional effects are identical and the conditioning variable is not a confounder.
Conditional hazard and hazard ratio	The hazard within a subset of the population defined by values of one or more variables, such as those with a particular value of a biomarker. A conditional hazard ratio is the hazard ratio comparing hazard between levels of a variable of interest (e.g. exposure or treatment) within this subset.
Frailty variable(s)	A variable or collection of unmeasured variables that affect when individuals experience the event of interest.
Hazard (function)	At a given time during follow-up, the hazard is the rate at which events are expected to occur in the next small interval of time. The hazard function gives the value of the hazard across all time values during follow-up.
Hazard ratio	The ratio of hazards at a given time during follow-up between two groups of individuals (often defined by levels of an exposure or treatment) still at risk for the event.
Marginal hazard and hazard ratio	The marginal hazard is the hazard function (across the subsets) in a given population. A marginal hazard ratio is a hazard ratio in the population.
Proportional hazards assumption	The assumption that a hazard ratio between two groups takes a common value across all times during follow-up.
Survival function	The survival function is a function of follow-up time. For a given time, its value is the proportion of individuals still event-free by that time.

## Introduction

Time-to-event outcomes are measured in many areas of health research. By far the most popular approach for analysing the relationships between time to event and covariates is Cox’s famous *proportional hazards* model [1, 2]. Cox’s model specifies how the hazard, the instantaneous failure rate among survivors at a given time, statistically depends on covariates. In a randomized trial, a Cox model with treatment group as the sole covariate assumes that the ratio of hazards between the two treatment groups remains constant over follow-up time: the proportional hazards assumption.

In recent years, some researchers have argued that HRs do not have a valid causal interpretation, even when the data arise from randomized trials, and even when the proportional hazards assumption holds [3–5]. In this article, we review these criticisms through a simplified example and describe how we think HRs should be interpreted.

## Hazards, hazard ratios, and proportional hazards models

Suppose we conduct a two-arm randomized trial. We let Z denote the randomized treatment group for a randomly selected individual, with Z=0 denoting randomization to the control arm and Z=1 denoting randomization to the new treatment, or ‘research’ arm. The hazard (event rate) at time *t* in the control group, h(t|Z=0), is defined as a mathematical limit such that


$$h(t|Z=0)\approx \;\frac{P(t\;\leq \;T<\;t+\;\Delta |T\;\geq \;t,Z=0)}{\Delta },$$

where T denotes the event time of a randomly chosen individual and Δ is small. As such, Δh(t|Z=0) is the expected proportion, among those still at risk at time t in the control arm, who will experience the event in the next Δ units of time. The hazard in the research arm is defined analogously. The (true) hazard ratio (HR) comparing the research group to the control group at a particular time *t* is then given by the ratio of the group specific hazards:


$$\mathrm{HR}(t;Z)=\frac{h(t|Z=1)}{h(t|Z=0)}.$$

A proportional hazards model with treatment as covariate specifies that


$$h(t|Z)=h_{0}(t)e^{\beta Z},$$

where $h_{0}(t)$ denotes the ‘baseline’ hazard. In this model, with treatment as the only covariate, this baseline hazard function specifies the hazard at each time point for the control group. Parametric proportional hazards models (e.g. the Weibull model) assume a particular form for the baseline hazard function $h_{0}(t)$, with its value specified by one or more parameters. In contrast, in Cox’s proportional hazards model, the researcher need not assume anything about how the baseline hazard $h_{0}(t)$ varies over time. The proportional hazards model with treatment as a covariate assumes that $\mathrm{HR}(t;Z)=e^{\beta }$, independent of *t*, which is referred to as the proportional hazards assumption. When the proportional hazards assumption is not deemed plausible, a common approach is to partition follow-up into nonoverlapping periods and fit an extended model which estimates a distinct HR parameter comparing treatment groups in each period [6]. Alternatively, methods exist for modelling the time-varying HR as a flexible smooth function of time, which are expected to yield more plausible and efficient estimates [6, 7].

Under the proportional hazards assumption, one interpretation sometimes given to $e^{\beta }$, including in some textbooks [8], is that it is the factor by which an individual’s hazard will be increased if they receive the research treatment rather than control. Such an interpretation as a common, individual-level effect is not generally justifiable due to the ubiquitous presence of so-called ‘frailty’—unmeasured factors that influence individuals’ hazards [3, 9]. Frailty similarly affects, the interpretation of the time-specific HR HR(t;Z) [4, 10].

## The effects of frailty on hazard

To understand the effects of frailty, we describe a simplified randomized trial example. Suppose that there exists an as-yet-undiscovered genetic risk factor *X* which affects survival. For half of the population X=0 and for the other half X=1. Within groups defined by the genetic risk factor *X* and randomized treatment *Z*, time to death is exponentially distributed with a constant hazard as specified by the values in Table 1.

**Table 1:** Hazard values h(t|X,Z) by genetic factor *X* and treatment group *Z* in idealized randomized trial.

Group	Hazard
X=1 , Z=0 (control)	0.5
X=1 , Z=1 (research)	0.25
X=0 , Z=0 (control)	0.1
X=0 , Z=1 (research)	0.05

Figure 1a and b shows the resulting *true* survival functions in the control and research arms respectively. Each sub-figure shows the survival functions S(t|X,Z) for the X=0 and X=1 individuals within that treatment arm. The survival functions give the probability that a randomly selected individual from the corresponding group survives to the given time. The figures also show expected numbers at risk displayed based on 500 individuals being randomized to each treatment arm, calculated assuming no individuals are lost to follow-up (censored). Since *X* is unmeasured, Fig. 1a and b also show what we can *actually* estimate: the treatment-group-specific overall survival function S(t|Z), which ignores or is marginal to *X*, represented by the thicker solid lines. These overall treatment arm-specific survival functions S(t|Z) are weighted averages of the respective X=0 and X=1 survival functions S(t|X=0,Z) and S(t|X=1,Z), with weights corresponding to the proportion without and with the genetic risk factor, here 0.5. These were derived analytically, with details available in the Supplementary Material.

**Figure 1:**
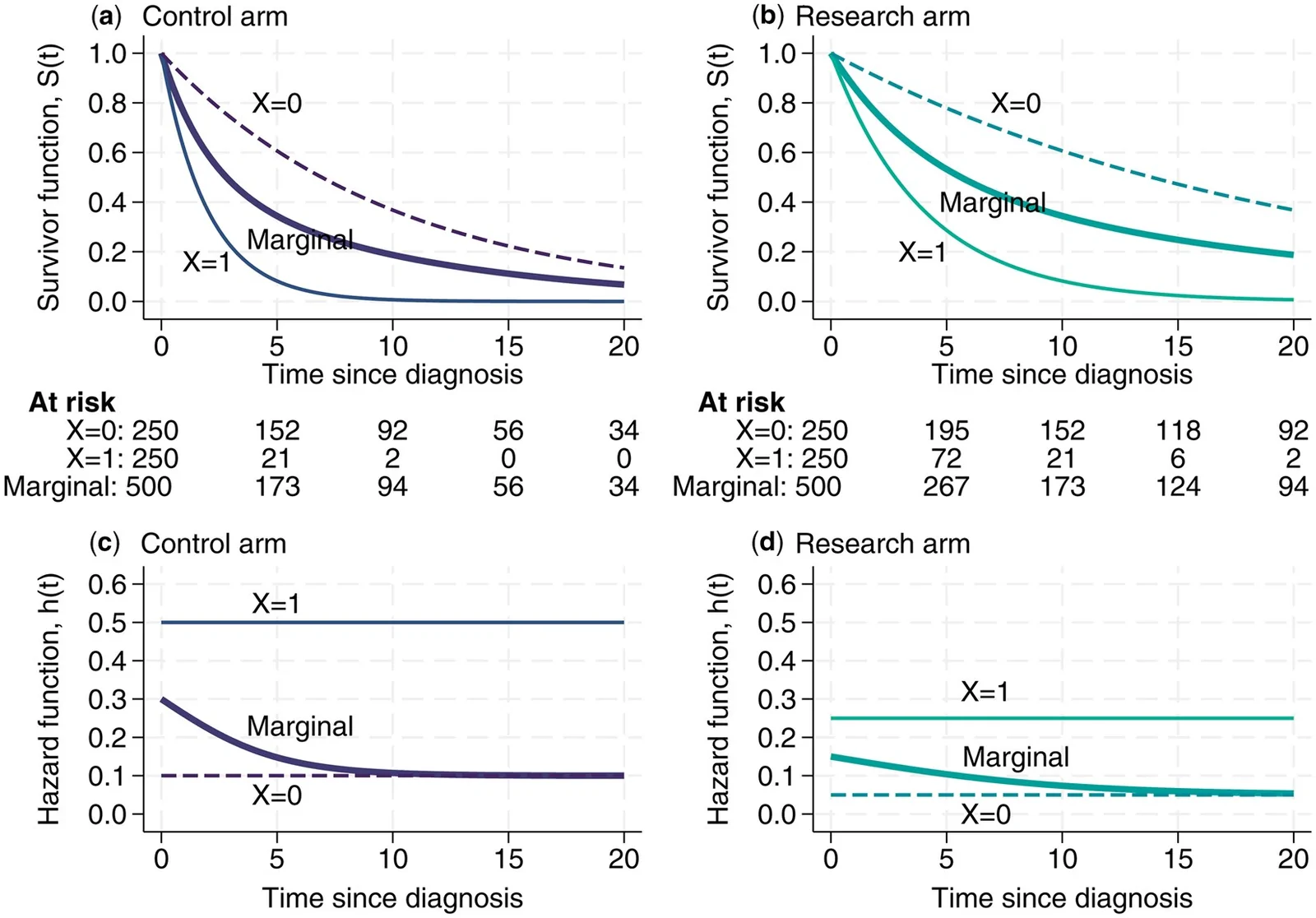
(a, b) Survival and (c, d) hazard functions by treatment group and unmeasured genetic risk factor *X* in idealized trial. (a, c), Control arm, (b, d), research arm.

Figure 1c and d shows the corresponding hazard functions conditional on treatment Z and X, h(t|X,Z), and the hazards functions marginal to X. In the control arm, because initially there are equal proportions of X=0 and X=1 individuals, the hazard marginal to *X*, h(t|Z=0) for early times in the follow-up is the average of the *X*-specific hazards 0.1 and 0.5. As time passes, the hazard h(t|Z=0) reduces, despite the hazard in groups defined by the genetic factor (i.e. h(t|X,Z)) remaining constant over time. This is because, as Fig. 1a shows, at later follow-up times, the survivors are dominated by X=0 individuals because they have lower hazard than those with X=1. Consequently, the hazard marginal to *X*, h(t|Z=0) increasingly resembles the hazard in the X=0 group. A similar phenomenon occurs in the research arm. This simple illustration shows that one should not generally interpret a hazard as a common hazard pertaining to each individual in the study, but rather as the event rate in the group of survivors at each time.

Now consider HRs comparing the research arm to the control arm. Recall from Table 1 that among individuals without the genetic risk factor (X=0), the HR comparing the research arm to the control arm is $\frac{h(t|X=0,Z=1)}{h(t|X=0,Z=0)}=\frac{0.05}{0.1}=0.5$, which does not vary with t (i.e. time-constant), and among individuals with the genetic risk factor (X=1), the (again time-constant) HR comparing the research arm to the control arm is $\frac{h(t|X=1,Z=1)}{h(t|X=1,Z=0)}=\frac{0.25}{0.5}=0.5$. Thus, conditional on or stratified by the risk factor *X*, there is a common, time-constant HR of 0.5. Recalling that *X* is unmeasured, the HR we can estimate is marginal to *X*. The true HR (marginal to *X*) comparing research to control arms at a given time, HR(t;Z), is the ratio of the true marginal hazards h(t|Z) in each arm and is shown in Fig. 2 (derivations for these are given in the Supplementary Material).

**Figure 2:**
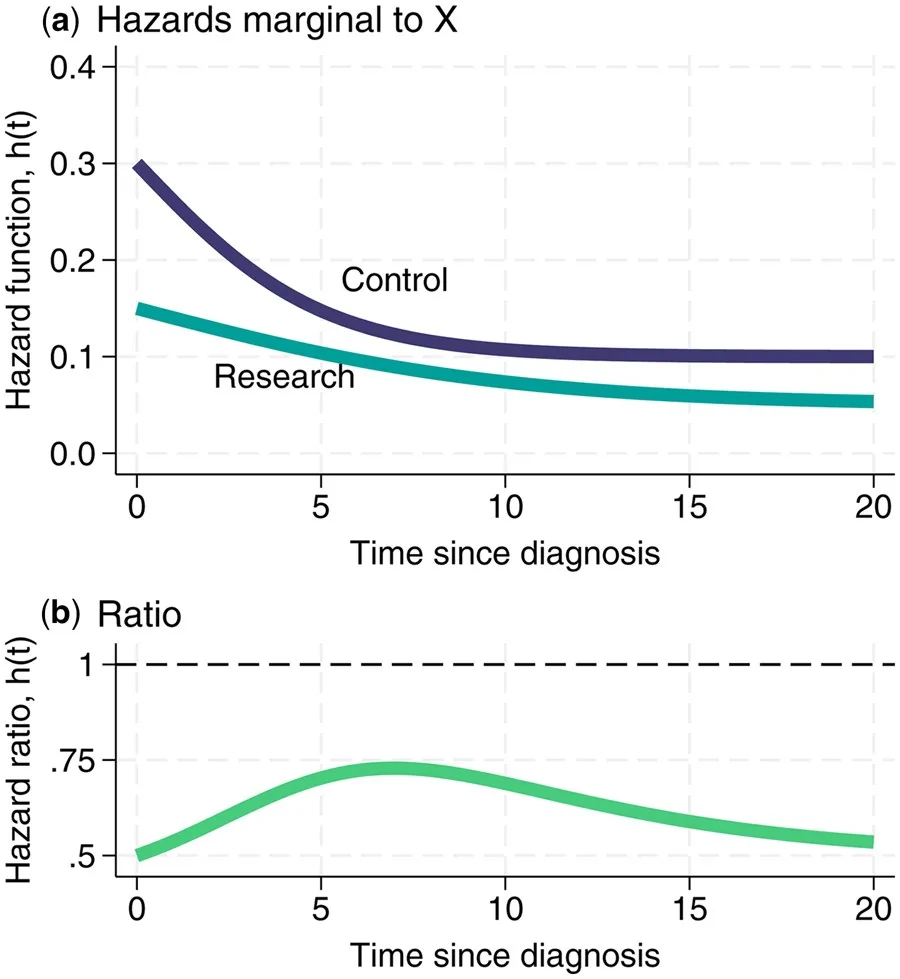
Hazards in each treatment arm, marginal to the genetic risk factor *X* (a), and their ratio (b).

Figure 2 shows HR(t;Z) is close to 0.5 early in the follow-up. This is because initially there is a 50:50 mix of X=0 and X=1 individuals among the survivors in both arms. As time progresses, however, HR(t;Z) begins to attenuate towards one, apparently indicating an attenuation of the treatment effect (as measured by HR) on the hazard of death. This is because in the middle stages of follow-up, among the surviving individuals in the research arm, a larger proportion have X=1 (e.g. 72/267 = 27% at *t* = 5) than among surviving individuals in the control arm (e.g. 21/173 = 12% at *t* = 5). Since individuals with X=1 have a higher hazard, this leads to a time-specific HR, HR(t;Z), that is closer to 1. Eventually, though, the survivors in the two treatment arms only consist of X=0 individuals, and so HR(t;Z) converges to the ratio of the X=0-specific hazards $\frac{h(t|X=0,Z=1)}{h(t|X=0,Z=0)}=\frac{0.05}{0.1}=0.5$.

Our simple example demonstrates that the value of HR(t;Z) depends on the two conditional (on *X*) HRs for treatment, together with the dynamically changing proportion of patients from each stratum in the two risk sets. We emphasize that the particular form in which HR(t;Z) varies over time (Fig. 2b) is specific to the setup we have considered, and other phenomena can occur, such as where HR(t;Z) converges steadily towards 1 [11].

The example, moreover, demonstrates an additional complexity—if the HR for treatment is constant over time conditional on certain variables (here *X*), it will in general not be constant over time marginal to these, even when there is no confounding [12]. As such, the proportional hazards assumption is unlikely to hold (at least exactly) in practice.

## Causal criticisms

Our simplified example demonstrates that the risk sets being compared at a given time *t* by HR(t;Z) are not (in general) similar in respect of baseline characteristics (e.g. *X* in our simplified example), due to differential depletion of susceptible individuals between treatment groups [4]. Because of this, HRs have been argued to be subject to selection bias [4], and not represent a valid causal effect of treatment, even in a randomized trial [3, 5, 13].

The imbalance in the distribution of baseline characteristics between risk sets can also be understood as a consequence of the fact that the HR involves conditioning on a collider [3, 14]. Figure 3a shows a directed acyclic graph, with the randomized treatment Z and frailty factor X. The node $S_{t}$ denotes whether an individual survives or not to time t and $S_{t+\Delta }$ whether they survive to time t+Δ. Treatment Z and frailty factor X affect both of these, and $S_{t}$ affects $S_{t+\Delta }$, since if an individual does not survive to time t, then that individual necessarily does not survive to time t+Δ. Treatment Z and frailty factor X are independent of each other due to treatment being randomized. As defined earlier, the hazard at time t considers the event rate among those who have survived to time t, i.e. it effectively conditions on the node $S_{t}$. $S_{t}$ is a so-called collider—arrows enter it from treatment Z and frailty factor X, and a consequence of this is to open a path between treatment Z and frailty factor X, as depicted by the dashed line in Fig. 3b. This means that treatment Z and frailty factor X are in general no longer independent among the survivors to time t.

**Figure 3:**
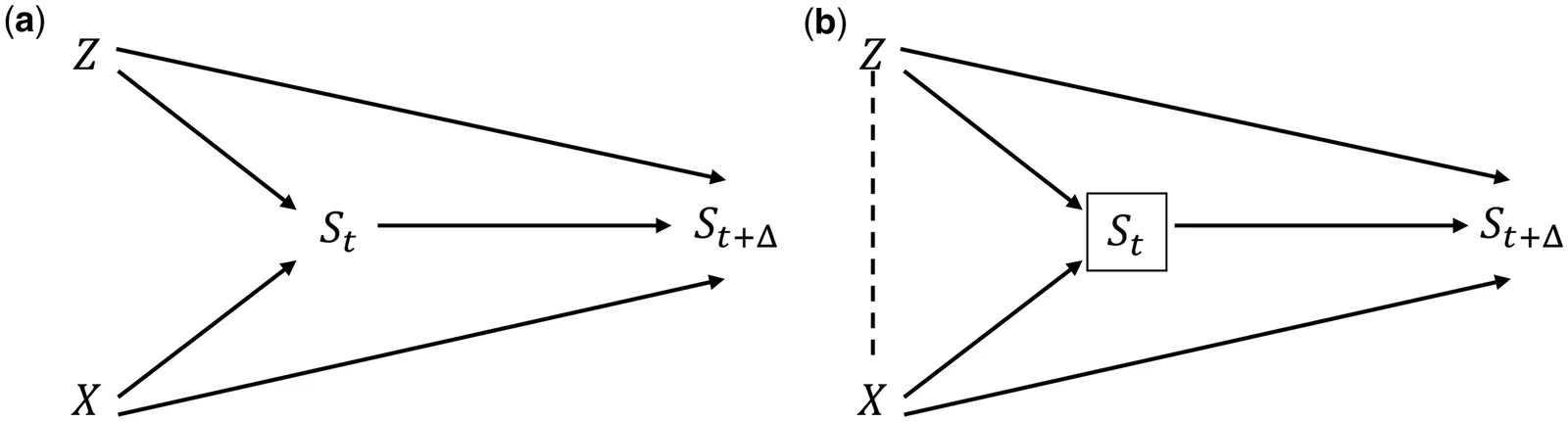
(a) Directed acyclic graph (DAG) showing causal relationships between randomized treatment (*Z*), frailty factor (*X*), survival to time *t* ($S_{t})$ and survival to time t+Δ ($S_{t+\Delta }$). (b) After conditioning on survival to time *t* ($S_{t})$, a path between treatment (*Z*) and frailty factor (*X*) is opened.

## How to (causally) interpret hazard ratios

As noted earlier, if HR(t;Z) is observed to vary over time t, various methods exist to estimate its time-changing value [6]. For concreteness, suppose HR(t;Z) takes value 0.5 at t=1. This would mean, if the population were assigned the research treatment, the hazard or rate of failure at time 1 among survivors to this time would be half what it would have been at time 1 had the population instead been assigned control treatment. Crucially, whenever the research treatment affects survival (compared to control treatment), the subset of the population that would survive to time *t* is systematically different in respect of baseline risk factors (such as *X*) under treatment *versus* control. This is a consequence of the earlier effects of the treatments and frailty factors on survival. We view HR(t;Z) as a causal population-level measure insofar as it captures how an observable population quantity (the hazard or rate of failure) would change if the population were given one treatment versus an alternative.

If instead HR(t;Z) is (approximately) constant over time (as assessed for example by examining a plot or test of Schoenfield residuals [15]), a Cox model including treatment arm would typically be adopted. In this case, the interpretation given above continues to hold, with now the common HR value applying for all times *t*. We note that, even in this case, it is possible (although arguably unlikely) for frailty factors to exist but in such a way that the HRs conditional on these variables $\left(\text{e.g.}\frac{h(t|X=x,Z=1)}{h(t|X=x,Z=0)}\right)$ vary with time, despite the fact that HR(t;Z) is constant [5]. As such, we emphasize that HR(t;Z) being constant or approximately constant over time does not by itself imply the absence of such frailty factors.

## Discussion

Hazards and contrasts of them are fundamental quantities in the analysis of time-to-event data and, rightly or wrongly, analyses of time-to-event outcomes are still almost always based on Cox’s proportional hazards model. Causal inference ideas, beginning from Hernán’s influential ‘Hazard of Hazard Ratios’ paper, have led to a fuller understanding of the complexities involved in the interpretation of HRs [4]. In light of criticisms of the HR from a causal perspective, our aim here was to elucidate the key aspects of these criticisms in a simple example and to describe how we think HRs can be legitimately interpreted in a causal sense.

Our example was deliberately contrived to discuss and visualize HRs: the event was common and the effect of frailty immodest (conditional HR of 5). As has been noted by others previously, the impacts are smaller when the event is rare or the frailty/unmeasured variables have modest effects [5, 11].

Randomized trial analyses using Cox models often adjust for a small number of baseline covariates to improve statistical power [16], while analyses of non-randomized studies may do so to adjust for confounding. If the model used is correctly specified, the resultant HRs for treatment can be interpreted as we have described, within subpopulations defined by the values of these covariates.

Given the complexities involved in correctly interpreting hazards and HRs, particularly when the HR is not constant over time, it may be preferable to quantify effects using alternative measures [17]. These include differences or ratios of survival or risk probabilities at clinically relevant landmark times, or contrasts of restricted mean survival to a given time horizon. These measures, in particular the differences or ratios of survival or risk probabilities, are easier to (correctly) interpret than the HR, can be defined without reference to modelling assumptions, and do not suffer from noncollapsibility [18, 19]. They nevertheless require choice of a landmark or horizon time. A further alternative is accelerated failure time models [20]. In settings where the proportional hazards assumption is materially violated, often no single number will be able to adequately summarize the differences in survival curves, and display of the survival curves themselves and multiple effect measures may be needed to most clearly communicate the differences [17].

## Supplementary Material

dyag228_Supplementary_Data

## Data Availability

No new data were generated or analysed in support of this research.

## Artificial intelligence

Claude was used to explore and discuss different definitions of ‘collapsible effect measure’.
