# Supplementary material for: Hazard ratios and their causal interpretation: challenges and guidance
Source: Int J Epidemiol. 2026 Oct 24;55(6):dyag228. doi: 10.1093/ije/dyag228 (PMC13695446; doi:10.1093/ije/dyag228)
Supplement: Supplementary file 1 [file dyag228_supplementary_data.docx]

**Supplementary material for** **‘How to interpret hazard ratios’**

**Jonathan W. Bartlett^1*^, Dominic Magirr^2^, Tim P. Morris^3^**

**^1^** Department of Medical Statistics, London School of Hygiene & Tropical Medicine, UK

**^2^** Statistical Methodology, Novartis Pharmaceuticals AG, Basel, Switzerland

**^3^** Statistical Methodology, Novartis Pharmaceuticals UK Ltd., London, UK

We derive expressions for the survival and hazard marginal to the frailty variable *X* in a given treatment group. We omit conditioning on the treatment group variable *Z* here to simplify notation. Assume that event times are exponentially distributed in the $X=0$ group with hazard $\lambda_{0}$ and in the $X=1$ group with hazard $\lambda_{1}$. Then the corresponding survival functions are given by

$$S\left( t | X=0 \right)=e^{-\lambda_{0}t}$$

$$S\left( t | X=0 \right)=e^{-\lambda_{1}t}$$

The survival function marginal to *X* is then given by

$$S\left( t \right)=P\left( T>t \right)=P\left( X=0 \right)P\left( T>t|X=0 \right)+P\left( X=1 \right)P\left( T>t | X=1 \right)$$

$$=P\left( X=0 \right)e^{-\lambda_{0}t}+P(X=1)e^{-\lambda_{1}t}$$

We now use the relationship between the hazard and the survival function, specifically that $h\left( t \right)=-\frac{d}{dt}\log S(t)$ to find the marginal hazard function. We have

$$\log S(t)=\log\left\{ P\left( X=0 \right)e^{-\lambda_{0}t}+P(X=1)e^{-\lambda_{1}t} \right\}$$

and so the marginal hazard is given by

$$h\left( t \right)=\frac{\lambda_{0}P\left( X=0 \right)e^{-\lambda_{0}t}+\lambda_{1}P(X=1)e^{-\lambda_{1}t}}{S(t)}$$
